# Mesenchymal stem cells correct haemodynamic dysfunction associated with liver injury after extended resection in a pig model

**DOI:** 10.1038/s41598-017-02670-8

**Published:** 2017-06-01

**Authors:** Hans-Michael Tautenhahn, Sandra Brückner, Christiane Uder, Silvio Erler, Madlen Hempel, Martin von Bergen, Janine Brach, Sandra Winkler, Franziska Pankow, Claudia Gittel, Manja Baunack, Undine Lange, Johannes Broschewitz, Matthias Dollinger, Michael Bartels, Uta Pietsch, Kerstin Amann, Bruno Christ

**Affiliations:** 1 Clinics of Visceral, Transplantation, Thoracic and Vascular Surgery, University of Leipzig, Liebigstraße 21, 04103 Leipzig, Germany; 2 Institute of Biology, Molecular Ecology, Martin-Luther-University Halle-Wittenberg, Hoher Weg 4, 06099 Halle, Germany; 3 Department of Molecular Systems Biology, Helmholtz Centre for Environmental Research, Permoser Strasse 15, 04318 Leipzig, Germany; 4 Institute of Biochemistry, Faculty of Biosciences, Pharmacy and Psychology, University of Leipzig, Brüderstraße 34, 04103 Leipzig, Germany; 5 Large Animal Clinic for Surgery, University of Leipzig, An den Tierkliniken 21, 04103 Leipzig, Germany; 6 Department of Medicine I, University Hospital Ulm, Albert-Einstein-Allee 23, 89081 Ulm, Germany; 7 Department of Anaesthesiology and Intensive Care, University Hospital Leipzig, Liebigstraße 20, 04103 Leipzig, Germany; 8 Department of Nephropathology, University Hospital Erlangen, Krankenhausstr. 8-10, 91054 Erlangen, Germany; 9 Clinics of Visceral and Vascular Surgery, University Hospital Jena, Erlanger Allee 101, 07747 Jena, Germany; 10 Klinik für Allgemein- und Viszeralchirurgie, Helios Park-Klinikum Leipzig, Strümpellstraße 41, 04289 Leipzig, Germany

## Abstract

In patients, acute kidney injury (AKI) is often due to haemodynamic impairment associated with hepatic decompensation following extended liver surgery. Mesenchymal stem cells (MSCs) supported tissue protection in a variety of acute and chronic diseases, and might hence ameliorate AKI induced by extended liver resection. Here, 70% liver resection was performed in male pigs. MSCs were infused through a central venous catheter and haemodynamic parameters as well as markers of acute kidney damage were monitored under intensive care conditions for 24 h post-surgery. Cytokine profiles were established to anticipate the MSCs’ potential mode of action. After extended liver resection, hyperdynamic circulation, associated with hyponatraemia, hyperkalaemia, an increase in serum aldosterone and low urine production developed. These signs of hepatorenal dysfunction and haemodynamic impairment were corrected by MSC treatment. MSCs elevated PDGF levels in the serum, possibly contributing to circulatory homeostasis. Another 14 cytokines were increased in the kidney, most of which are known to support tissue regeneration. In conclusion, MSCs supported kidney and liver function after extended liver resection. They probably acted through paracrine mechanisms improving haemodynamics and tissue homeostasis. They might thus provide a promising strategy to prevent acute kidney injury in the context of post-surgery acute liver failure.

## Introduction

Extended partial liver resections of more than 70% render the residual organ with a high regenerative demand. The surgical risk *per se* is further augmented by accompanying kidney dysfunction^1, 2^ due to hepatic ischaemia caused by the Pringle’s manoeuvre during partial liver resection. This causes haemodynamic turbulences followed by reduced lung, heart and kidney perfusion, the latter eventually provoking acute kidney injury (AKI)^3^. A similar mechanism applies to the development of the hepatorenal syndrome in acute-on-chronic liver failure, which has led to the concept of classifying renal failure associated with cirrhosis within the framework of the acute kidney injury network^4–6^.

AKI is characterized by a defined rapid reduction of the glomerular filtration rate (GFR) evidenced by less urine production and the elevation of serum creatinine levels^7^, criteria which have also been applied to classify AKI in patients with liver cirrhosis^5, 8, 9^. The validity of creatinine to assess kidney function, however, remains questionable, because it changes late in AKI and is affected by individual muscle mass, diet, age and medication. Recently, interleukin-18 (IL-18), kidney injury molecule 1 (KIM-1) and neutrophil gelatinase-associated lipocalin (NGAL) were defined early prognostic markers of AKI^10^. NGAL is considered a versatile biomarker of AKI in the context of liver cirrhosis^11–13^. It is produced by several organs including kidney and liver, and is significantly elevated in the kidneys upon ischaemic insult or toxic injury. However, NGAL is also elevated in serum and in urine during systemic inflammation, and therefore has to be considered carefully related to kidney injury^14, 15^. It was though discussed, whether urinary NGAL was increased due to the inflammatory response associated with sepsis or resulted from early kidney damage^16^. Its appearance in the urine indicates the impairment of re-uptake due to acute damage of the proximal tubule^10^. In line, early AKI induced by ischaemia and reperfusion, hepatorenal syndrome or by drug toxicity (e.g. calcineurin inhibitor) led to the detachment of proximal tubular epithelial cells from their basal membrane as the consequence of the loss of polarity and brush border membrane destruction as well as disaggregation of the actin cytoskeleton^17, 18^. At later stages, tubular necrosis, the drop of GFR, the rise in serum creatinine, as well as kidney tissue and systemic inflammation are hallmarks of progressing AKI^19^.

Mesenchymal stem cells (MSCs) act pro-regenerative, anti-inflammatory and vasoprotective, all potential features supporting kidney recovery and function to ameliorate AKI as shown in *in vitro* and in small animal models of AKI^20, 21^. Furthermore, it has been shown that MSCs ameliorated acute liver damage after extended liver resection in the rat and improved survival of the animals by paracrine mechanisms^22, 23^. Clinically, donor-derived MSCs suppressed alloreactive kidney graft rejection substantiating the anti-inflammatory and immune-regulatory features of MSCs also in humans^24^. Currently, it is unclear, whether haemodynamic changes like splanchnic vasodilation causing the fall in systemic vascular resistance and the decrease in arterial pressure, and thus contributing to the hyperdynamic circulation as observed in the hepatorenal syndrome associated with liver cirrhosis^25^, may also apply to acute kidney injury after extended liver resection. So far, limited and inconsistent clinical data are available regarding AKI in response to extended liver resection. Therefore, we investigated the occurrence of acute kidney damage after extended liver resection in the pre-clinically relevant pig model of 70% liver resection, the impact of MSC application on the attenuation of kidney damage, and finally tried to identify paracrine factors and pathways potentially involved in MSC action.

## Results

### Liver injury after extended resection

**Figure 1:**
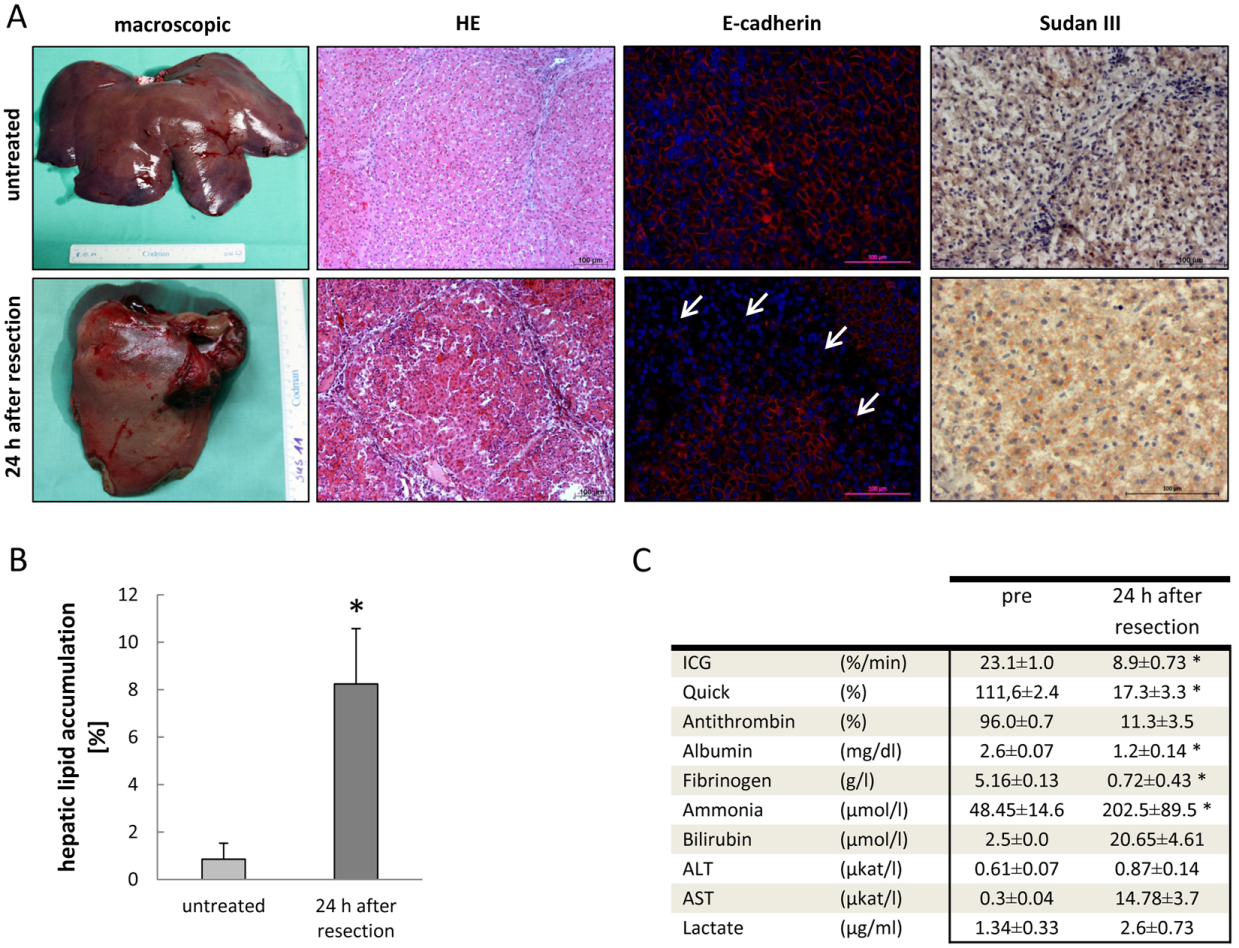
Summary of data indicating liver damage after extended resection. (**A**) Macroscopic and histological signs of liver damage. Macroscopically, livers displayed a paler aspect after resection as compared with untreated animals. This was due to the increase in lipid accumulation (Sudan III and (**B**)). Impairment of tissue integrity after resection was obvious by the HE stain and the distortion of E-cadherin expression (red). While in sham animals E-cadherin is expressed ubiquitously, large regions are void of expression (white arrows) in livers of resected animals. Functional data of liver injury are summarized in (**C**). *Values are significantly different from controls at the p < 0.05 level using *t*-tests for depending samples.

To confirm severe liver damage after extended resection, histological parameters indicative for liver impairment were investigated in livers of sham animals and animals 24 h after resection. While the macroscopic aspect did not show obvious signs of tissue damage, the HE stain yielded deterioration of the liver parenchyma with widened sinusoids and damaged hepatocytes in the resected animals (Fig. 1A, HE). We have shown previously that cell adhesion contacts were disrupted during liver injury by the impairment of E-cadherin expression^26^. This was corroborated in the present study showing that the integrity of E-cadherin expression was disturbed in resected as compared with sham animals (Fig. 1A, E-cad). As a sign of progression towards post-surgery acute liver failure, we recently identified increasing lipid accumulation in rat livers after 90% partial hepatectomy^22^. In the present study staining of lipids with Sudan III revealed significantly higher lipid content in the livers of animals after liver resection as compared to sham animals (Fig. 1A, Sudan III). It was increased roughly by 4-fold over sham animals as calculated by image quantification using Image J (Fig. 1B).

To further substantiate liver damage by the partial resection, hepatic functional parameters were measured in the serum before and 24 h after resection. The indocyanine green clearance, representing phase III detoxification, decreased from 23.1 ± 1.0 to 8.9 ± 0.73%/min. Blood coagulation was affected as demonstrated by the 6-fold decrease of the Quick value. Plasma protein synthesis was generally impaired as shown by the decrease of antithrombin, albumin, and fibrinogen serum levels. Serum ammonia and bilirubin increased by 4- and 8-fold, respectively, indicating loss of substantial hepatic functions. Finally, transaminases increased indicative for hepatocyte damage. Lactate was doubled suggesting an overall organ malperfusion (Fig. 1C). In addition, serum sodium concentrations were lowered significantly (see below), which is very often associated with chronic liver diseases^4^. We furthermore raised data from transcriptomic analysis of samples after liver resection in the absence and presence of BM-MSCs. Since clustering into pathways is more reliable than focusing on single genes, we present here the results from the data analysis by Ingenuity Pathway Analyser (IPA). In this approach, several pathways related to severe liver damage after resection were detected with statistically significant scores. First, the pathways of EIF2 signaling and regulation of eIF4 and p70S6K signaling were clearly induced (−log (pvalue) = 12.5 and 5.36), respectively. Since EIF 2 controls protein synthesis, this pathway is part of the proliferation in response to liver resection^27^. The induced pathway of “Remodelling of Epithelial Adherens Junctions” (−log (p-value) = 3.66) indicates broad tissue remodeling in the liver samples. In addition, genes belonging to the aryl hydrocarbon pathway comprise those that are linked to oxidative stress (−log (p-value) = 2.52). Furthermore, the pathway of IL-1 signaling, including the cytokine itself as well as prominent parts of the NF-kappa B pathway indicate the relevance of inflammatory processes in the liver after extended resection. Thus, major functional features of the liver were impaired, and we concluded that the method of extended liver resection as described by Arkadopoulos^28^, which we applied in our study, caused severe liver damage.

### Acute kidney damage after extended liver resection and improvement by pBM-MSCs

**Figure 2:**
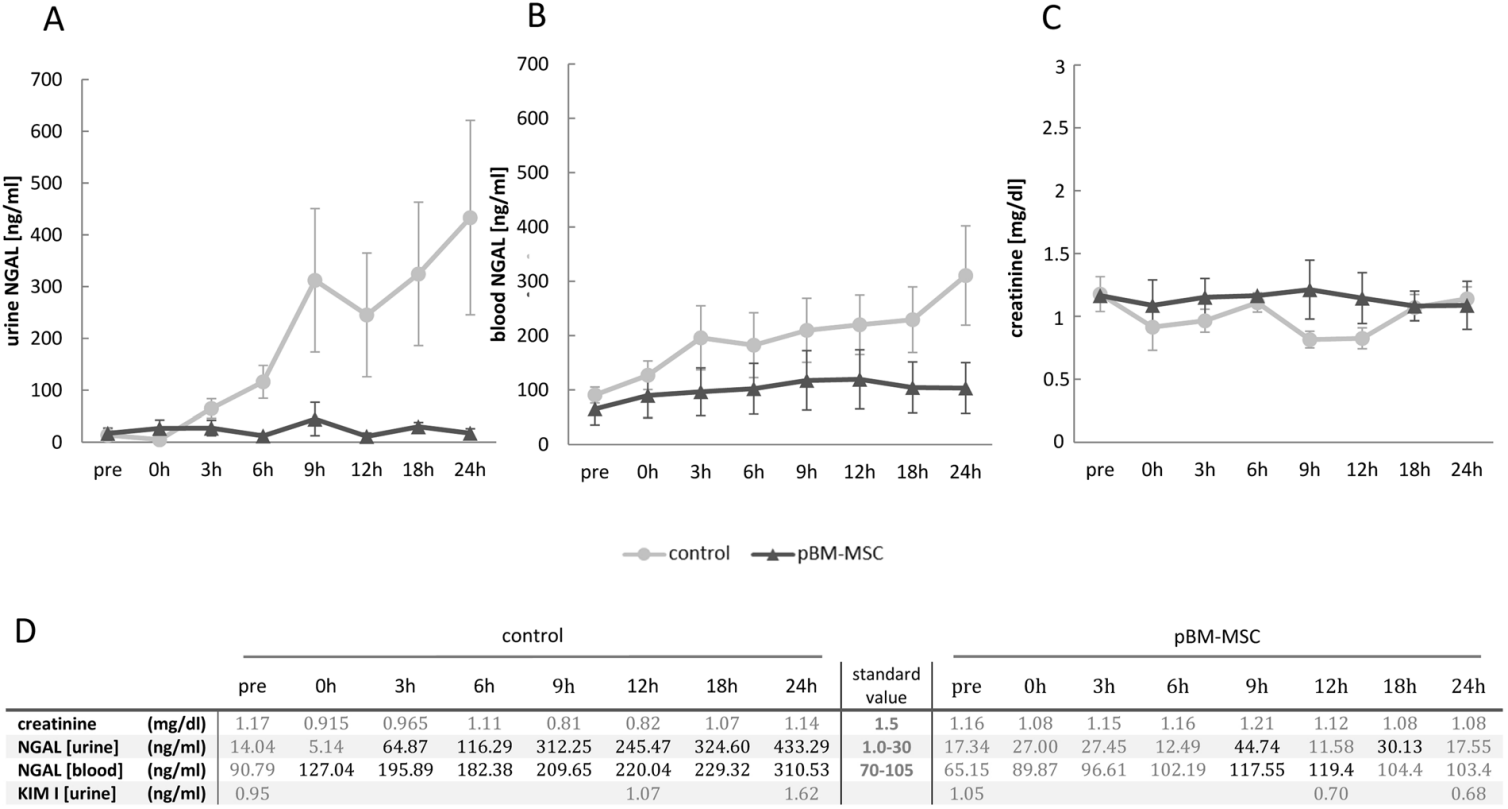
Changes in NGAL and creatinine during 24 h following extended liver resection. NGAL (ng/ml) in urine (**A**) and serum (**B**) of control animals and in animals treated with pBM-MSC. (**C**) Creatinine (mg/dl) levels in the urine of control and pBM-MSC treated animals. (**D**) Elevation over standard values of mean creatinine and NGAL as well as KIM-1 values in serum and urine in the control and the pBM-MSC group is indicated by bold figures. Statistically significant differences according to the Bonferroni post-hoc test following general linear model.

The surgical procedure applied here considerably disturbs the haemodynamic homeostasis, which is known to promote AKI. Accordingly, the early marker of AKI, NGAL, increased in urine and blood serum significantly over time (GLM: F = 2.35, *p* = 0.034). After 24 h, it was elevated by about 30-fold in the urine (Fig. 2A and D, left) and by about 3-fold in the blood (Fig. 2B and D, left). Values were higher than standard values at nearly each point in time measured. Porcine BM-MSCs prevented the increase in NGAL in urine (GLM: F = 19.38, *p* < 0.0001) and blood serum (GLM: F = 14.71, *p* = 0.0003), and elevation over standard values was hardly observed (Fig. 2A,B and D, right). Animals were treated with antibiotics during the experiment. Hence it may be ruled out that bacterial infection caused the increase in NGAL.

Another sensitive early marker of kidney injury due to distortion of proximal tubular integrity is kidney injury molecule-1 (KIM-1). The membrane protein is released into the urine by shedding and has been demonstrated, both pre-clinically and clinically, to be elevated in kidney injury of multiple different etiologies^29^. KIM-1 was measured pre-surgery and at the time points 12 and 24 h after liver resection. Albeit not significantly, it was increased after liver surgery. The increase was prevented by the treatment with pBM-MSC (Fig. 2D). IL18, another potential early prognostic marker of AKI^29^, was unchanged both in serum (as quantified by ELISA, not shown) and kidney (see below for quantification of expression in the kidney). To further substantiate that kidney function was severely impaired, urine production was quantified. Over the 24 h observation period, it amounted to 11.8 ml/h × kg in sham animals, which was significantly reduced to 3.3 ml/h × kg in control animals, and reconstituted in part to 7.9 ml/h × kg by pBM-MSC treatment. Creatinine concentrations in the serum did not change during the 24 h observation period (Fig. 2C), which is consistent with previous data showing no significant increase in creatinine during 14 days after 50–60% liver resection in piglets^30^. In addition, it is known that creatinine is a late indicator of AKI lagging behind the increase in NGAL, and which may be also influenced by factors like age, gender, weight or muscle mass^11^.

**Figure 3:**
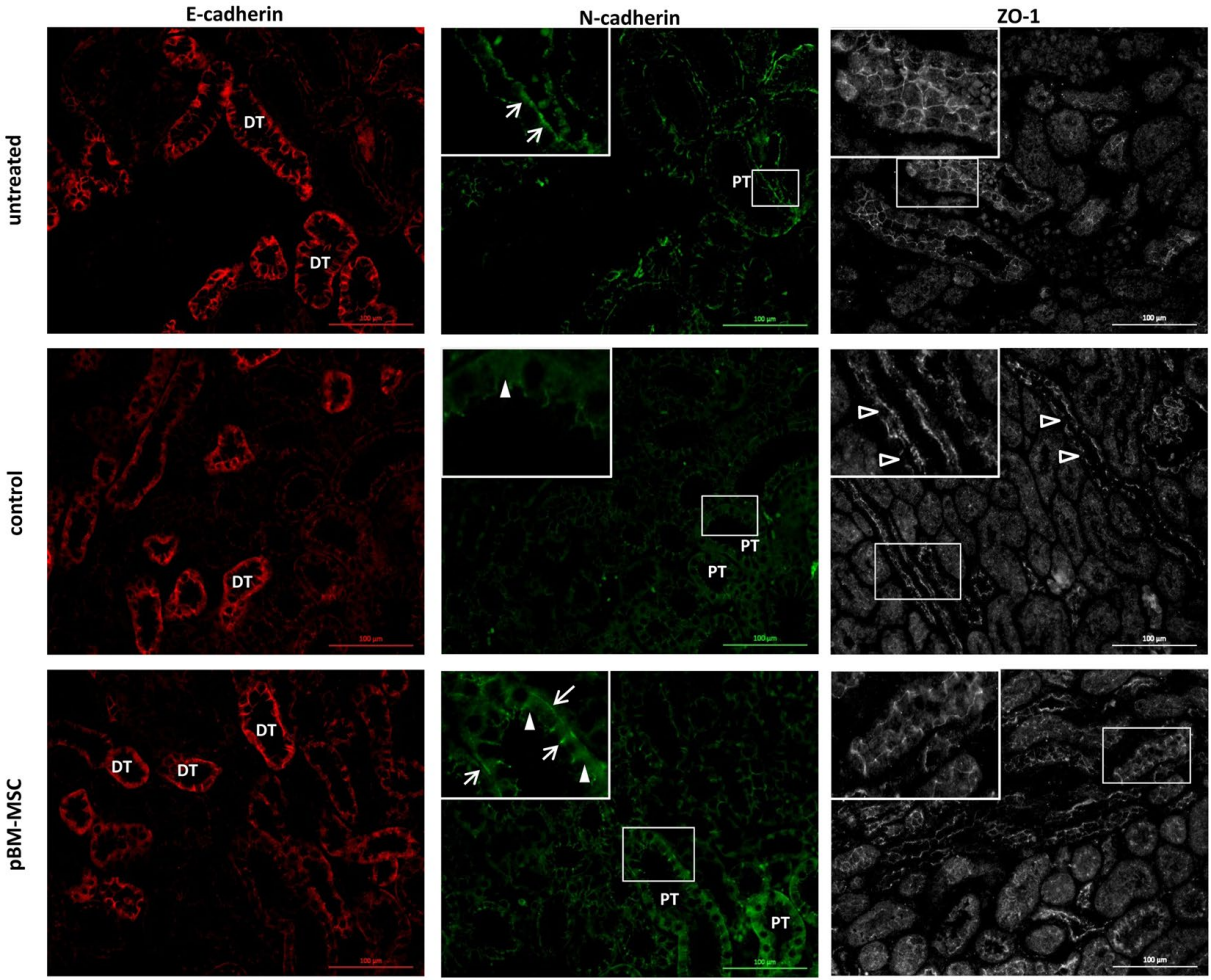
Immunostaining of E-cadherin, N-cadherin and ZO-1 in kidneys of untreated animals and after extended liver resection. E-cadherin (left panels, red), N-cadherin (middle panels, green) co-stains and ZO-1 (right panels) were detected by immunofluorescence in kidney tissue of untreated animals (top) and of animals after partial hepatectomy without (control, centre) or with application of pBM-MSC (bottom). Original magnification: 200x; DT = distal tubule; PT = proximal tubule. Arrows and solid arrowheads indicate membrane and cytoplasmic localization of N-cadherin, respectively. Open arrowheads point to discontinuous expression of ZO-1 after liver resection.

**Figure 4:**
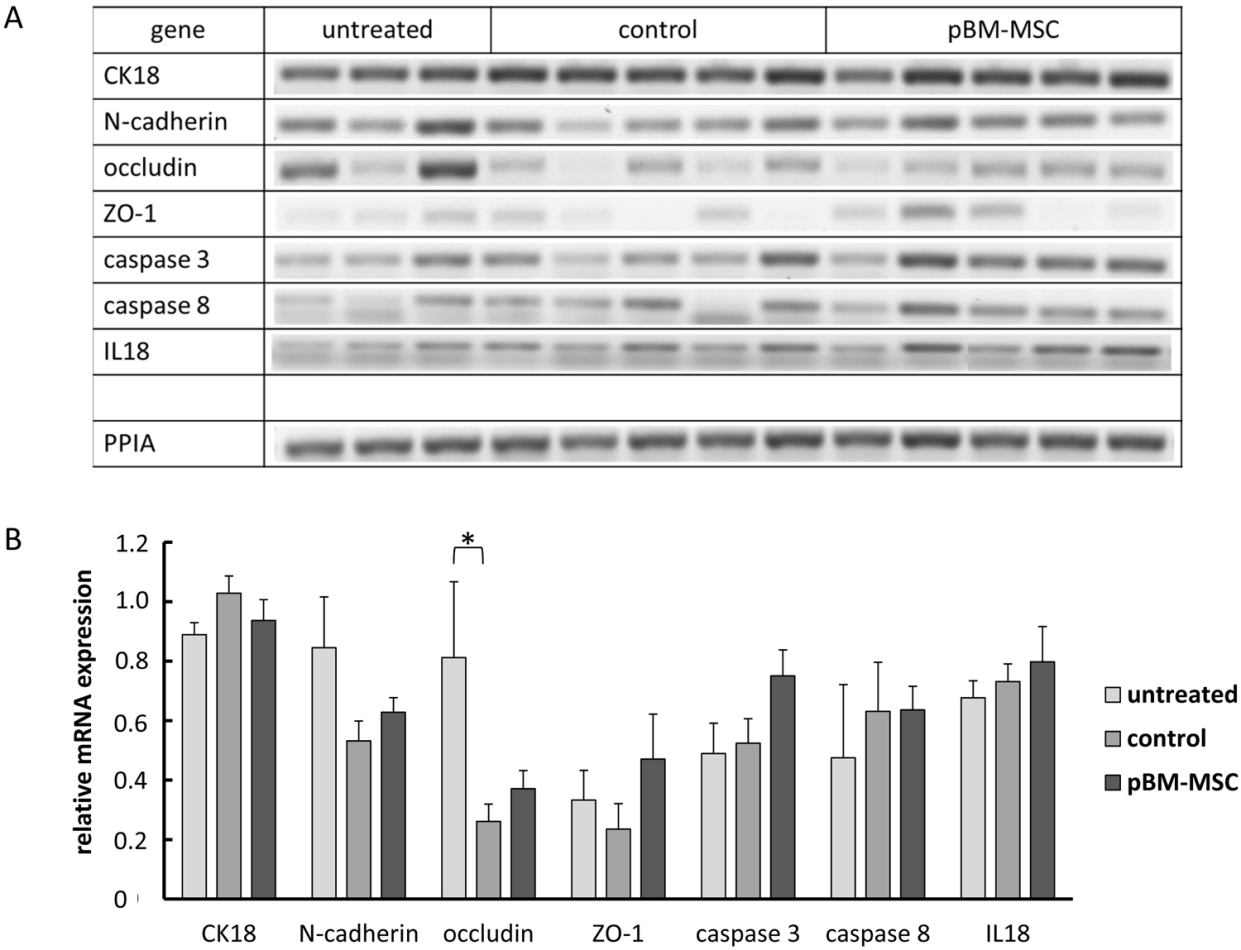
Quantification of CK18, cell contact proteins N-cadherin, occludin, ZO-1, and caspases as well as IL18 by RT-PCR. Expression levels were determined by quantification of PCR products after electrophoretic separation (**A**) using the Image J software (**B**). PPIA (Peptidylprolyl Isomerase A) was used for standardization of loading.

AKI induced by ischaemia or toxins was characterised by the impairment of kidney function due to the loss of tubular epithelial polarity^31^. Tissue integrity was checked by the immunohistochemical detection of the adhesion junction molecules E- and N-cadherin. Co-staining of E- and N-cadherin allows for the discrimination between proximal and distal tubular cells, because E-cadherin is the major adherens junction protein in distal tubules and N-cadherin in both distal and proximal tubules^32^. This distribution was confirmed in the kidneys of untreated animals with N-cadherin mainly localised to the plasma membrane (Fig. 3, top left and middle). After extended liver resection, N-cadherin was apparently lower in the control group and localised rather in the cytoplasm than in the membranes, whereas E-cadherin remained unaffected (Fig. 3, centre left and middle). In the pBM-MSC group, N-cadherin was detected again at the cell surface of proximal tubular cells, but also still in the cytoplasm (Fig. 3, bottom left and middle). The decrease of N-cadherin expression and its cytoplasmic re-distribution consecutive to extended liver resection might indicate tubular epithelial damage, which was ameliorated by pBM-MSC treatment. To confirm this assumption, the tight junction protein ZO-1 was detected immunohistochemically, which has been demonstrated to re-distribute from the plasma membrane to the cytoplasm after kidney ischemia-reperfusion injury^33^. In untreated animals, ZO-1 expression was continuously localised to the plasma membrane (Fig. 3, top right). This continuum was disrupted after liver surgery and re-constituted at least in part by the treatment with pBM-MSCs (Fig. 3, centre and bottom right). Thus, MSCs seemed to protect the epithelial integrity of proximal tubule cells from damage after extended hepatectomy. The amounts of cell adhesion molecule N-cadherin, of tight junction proteins ZO-1 and occludin as well as of the intermediary filament protein CK18 were determined by the quantification of RT-PCR products after electrophoretic separation. Occludin was significantly down-regulated by extended liver resection, whereas N-cadherin and ZO-1 were lower by trend albeit not significantly. There was a tendency of up-regulation again by pBM-MSC treatment; this, however, was not significant in either case. CK18 remained unchanged under each condition (Fig. 4A,B). This corroborated the immunohistochemical results shown in Fig. 3, which suggested that both the reversible membrane-cytoplasm re-distribution and the transcriptional down-regulation of cell contact proteins contributed to the disturbance of epithelial integrity induced by extended liver resection and to its amelioration by the stem cell treatment.

Expression levels of caspases 3 and 8 were not changed indicating that the changes in cell contact proteins did not necessarily induced ample apoptosis, at least during the 24 h observation period. This assumption is supported by the finding that no obvious tissue damage was detected in the kidney by staining with HE (not shown).

### Improvement of blood pressure homeostasis by pBM-MSCs

**Figure 5:**
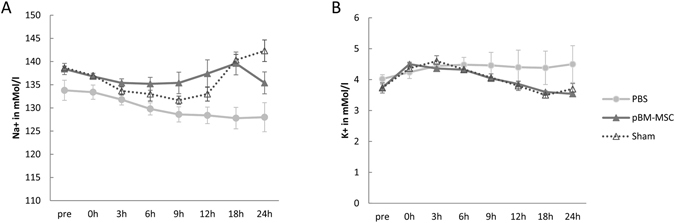
Changes in blood electrolytes during 24 h after extended liver resection. (**A**) Blood sodium concentration, and (**B**) blood potassium concentrations in control animals (dots), animals treated with pBM-MSC (solid triangles) and sham animals without liver resection (open triangles). Statistically significant differences according to the Bonferroni post-hoc test following general linear model.

A major complication in patients with advanced cirrhosis and hepatorenal syndrome is the decrease in organ perfusion due to peripheral vasodilation^34^. This might be compensated in part by systemic vasoconstriction mediated by the renin-angiotensin system in an attempt to maintain blood pressure homeostasis. Indeed, after partial liver resection, aldosterone was increased from 83 ± 0 pmol/l to 353 ± 50.4 pmol/l as compared with sham animals, but was lowered again to 121 ± 29.8 pmol/l after treatment with pBM-MSCs (GLM: F = 11.65, *p* = 0.002). Aldosterone secretion is a main target of angiotensin action and as a sign of pathologically elevated aldosterone levels and kidney damage, sodium excretion in the kidney is impaired. Consecutively, this leads to excessive water retention, ascites and hyponatraemia due to hypervolaemia. In the pig, standard sodium concentrations are in the range of 135–145 mmol/l. After extended liver resection, they decreased continuously over time from 133.8 ± 2.18 to 128 ± 3.13 mmol/l at 24 h post-surgery. Treatment with pBM-MSC corrected hyponatraemia significantly (p = 0.0001) resulting in the restoration of sodium serum levels to values of 135.4 ± 2.36 mmol/l at 24 h post-surgery, which was in the range of sham-operated animals (Fig. 5A). The lack of adequate aldosterone action due to acute kidney injury is also reflected by the decrease of potassium excretion in the kidney and consecutive hyperkalaemia. Reference serum values of potassium in the pig range between 3.6–5.2 mmol/l. In control animals, values increased over time from 4.02 ± 0.14 mmol/l up to 4.5 ± 0.6 mmol/l at 24 h after surgery. They were significantly lower after treatment with pBM-MSCs reaching 3.54 ± 0.04 mmol/l 24 h after surgery, which was in the range of values in sham-operated animals (Fig. 5B). Together these results underline the conclusion that the treatment of pigs with pBM-MSCs after extended liver resection corrected all signs of AKI measured here, and thus obviously protected the kidney from damage.

**Figure 6:**
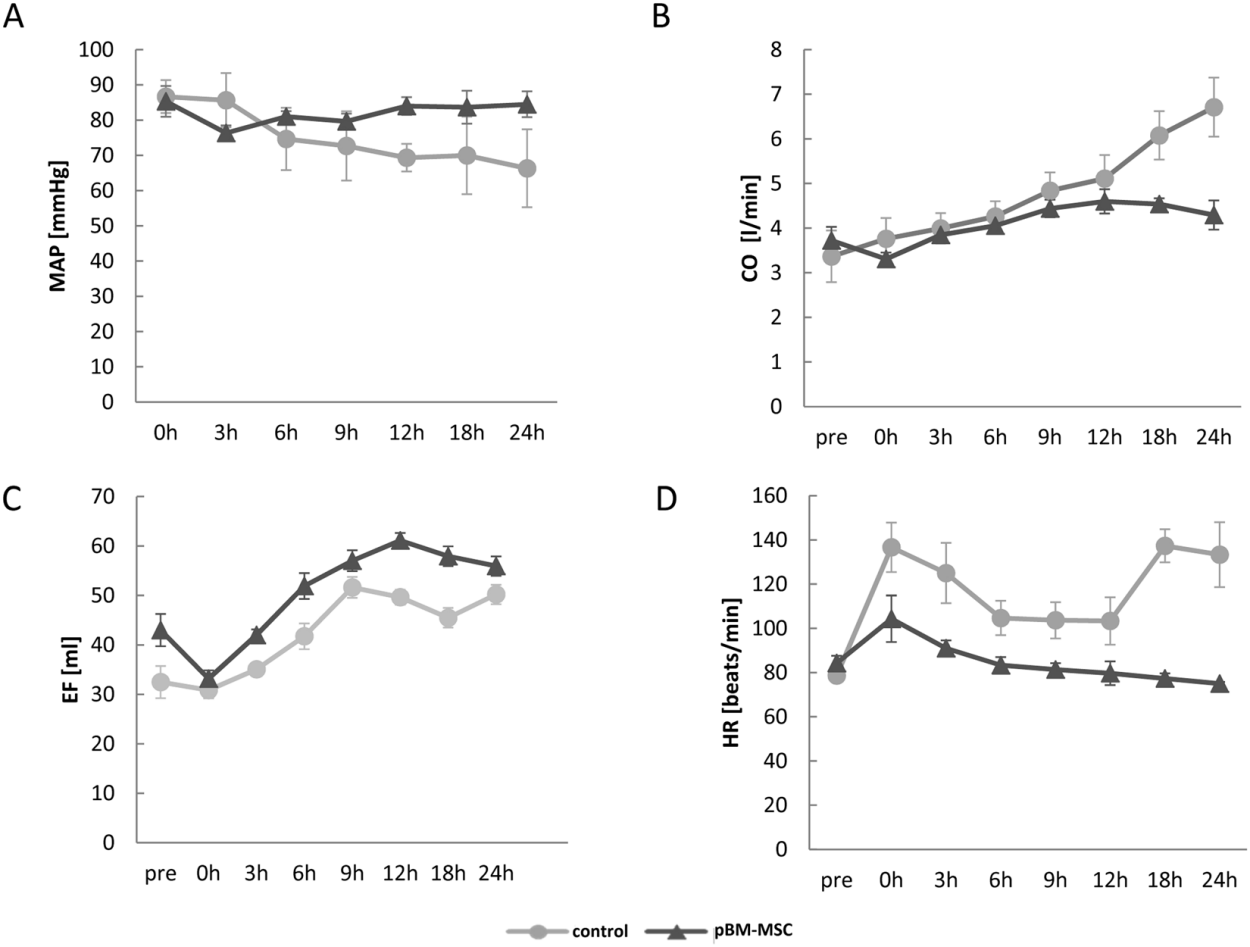
Changes in haemodynamic parameters during 24 h after extended liver resection. (**A**) Mean arterial pressure (MAP), (**B**) Cardiac output (CO), representing the product of the ejection fraction (EF) shown in (**C**) and the heart rate (HR) shown in (**D**) in control animals (dots) and animals treated with pBM-MSC (triangles). Statistically significant differences according to the Bonferroni post-hoc test following general linear model.

From these data it might be anticipated that blood pressure homeostasis was severely impaired after liver resection. This might be estimated from the determination of the mean arterial pressure (MAP). A drop of the MAP below 60 mmHg eventually leads to insufficient organ perfusion. After extended liver resection, the MAP was more stable after pBM-MSC application as compared to control animals, which displayed a time-dependent drop of the MAP near to or below the critical value of 60 mmHg (Fig. 6A). The heart may compensate for the decrease in vascular resistance by increasing the cardiac output. Thus, the MAP is proportional to the cardiac output (CO) and the total peripheral resistance (TPR). The CO is proportional to the ejection fraction and the heart rate. CO was rising significantly over time in both the control and the pBM-MSC group (GLM: F = 7.89, *p* < 0.0001), but to a significantly higher degree in the control than in the pBM-MSC group (GLM: F = 12.01, *p* = 0.001) (Fig. 6B). Between 12 and 24 h, the CO was increasing in the control group, while it kept constant in the pBM-MSC group. This was mainly due to the increase in the heart rate (GLM: treatment-F = 77.39, *p* < 0.0001; time-F = 4.02, *p* = 0.0023) (Fig. 6D), which was significantly higher in the control than in the pBM-MSC group. The ejection fraction increased in both groups over time (GLM: treatment-F = 17.8, *p* = 0.0002; time-F = 11.22, *p* < 0.0001) (Fig. 6C). Thus, despite the increase in CO, the MAP decreased in control animals indicative for a consistently low TPR due to massive peripheral vasodilation. *Vice versa*, MSC treatment obviously supported the maintenance of the peripheral blood pressure, thus protecting organs, including the kidneys, from mal-perfusion. This conclusion is also corroborated by the significant decrease of lactate from 2.6 ± 0.73 µg/ml in control to 1.12 ± 0.10 µg/ml in MSC-treated animals indicating the improvement of overall organ oxidative metabolism by the stem cell treatment.

### Paracrine mechanisms of pBM-MSCs

**Figure 7:**
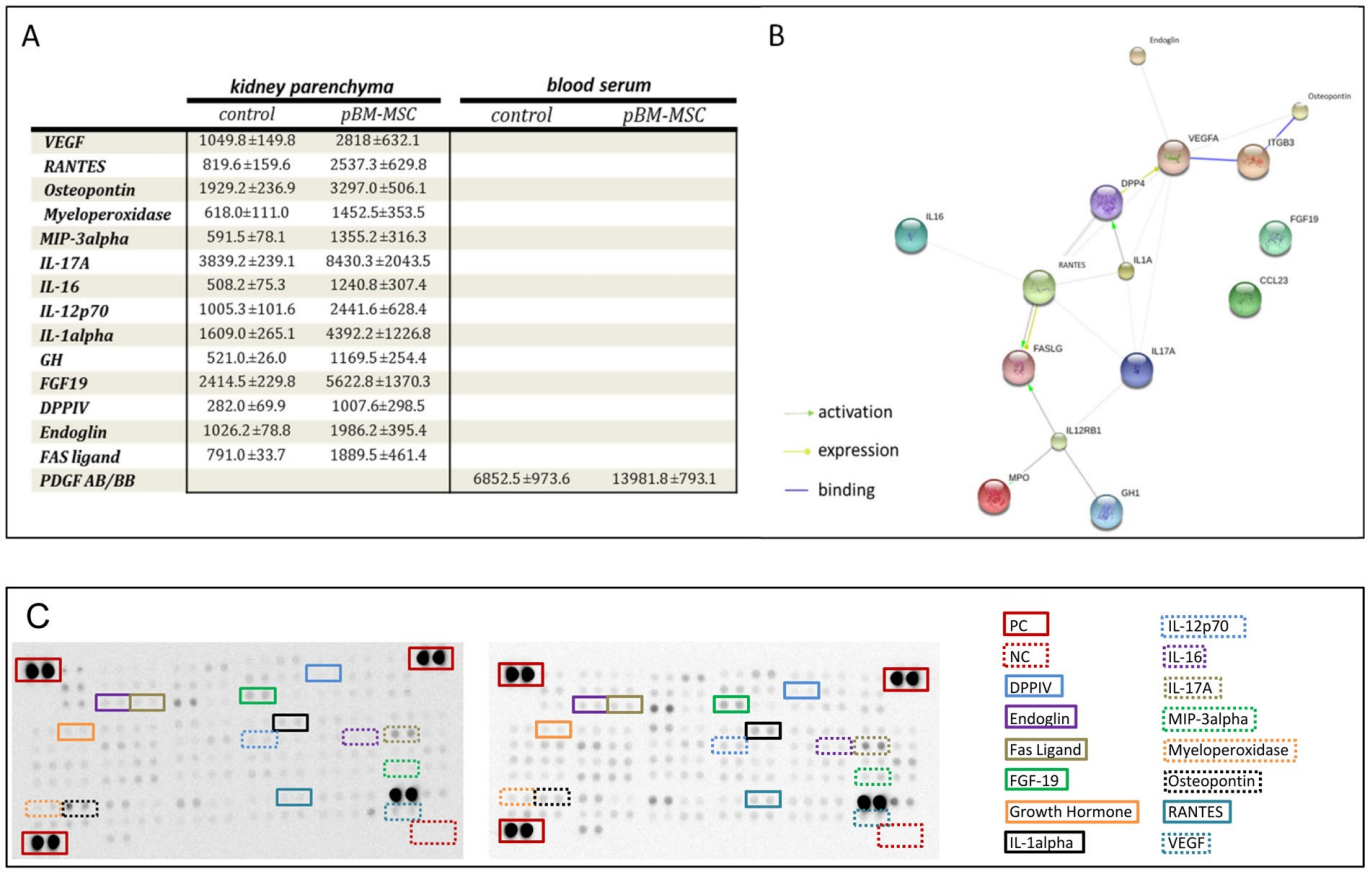
Increase in secretory proteins in blood serum and kidney parenchyma induced by pBM-MSC after extended liver resection. (**A**) Summary of proteins significantly (Student´s *t*-test; *p* < 0.05) elevated after pBM-MSC treatment in kidney tissue and blood serum. (**B**) Graphical visualization of predicted interactions between proteins elevated in kidney tissue by pBM-MSC using the STRING platform. (**C**) Representative Protein profiler arrays after detection of serum proteins from control (left) and MSC-treated (right) animals. PC = Positive control; NC = Negative control; DPP-4 = Dipeptidylpeptidase-4; FGF19 = Fibroblast Growth Factor 19; GH = Growth Hormone; IL = Interleukin; MIP-3alpha = Macrophage Inflammatory Protein 3 alpha; PDGF = Platelet-Derived Growth Factor; RANTES = Regulated on Activation, Normal T Cell Expressed and Secreted; VEGF = Vascular Endothelial Growth Factor.

Pathways potentially involved in the amelioration of acute kidney damage by pBM-MSCs were evaluated by determination of changes in cytokines and growth factors in blood serum and in kidney tissue of animals in the control and pBM-MSC groups. PDGF in the serum and 14 proteins in the kidney were significantly (*t*-test; *p* < 0.05) elevated in the pBM-MSC as compared to the control group (Fig. 7A). Graphical illustration by using the STRING database predicted potential interactions between these mediators (Fig. 7B). Accordingly, DPPIV might be activated in the kidney by IL-1A, thus impacting on the expression of VEGF and interacting with RANTES. RANTES and IL-12p70 might activate FAS ligand, a potential regulator of immune responses and apoptosis. Furthermore, pro-inflammatory or metabolically active cytokine interactions of IL-17A, IL-16 and FGF-19 were detected corroborating their known involvement in kidney inflammation and repair^35^. To identify regulatory networks, the mediators significantly elevated by pBM-MSC treatment were subjected to the pathway analysis by using the DAVID database. Associated pathways comprised immune system and inflammation regulatory networks like the cytokine-cytokine receptor interaction and Toll-like receptor signaling, pathways involved in the regulation of growth and tissue homeostasis like melanoma, focal adhesion, oncological pathways in cancer and MAPK signaling pathways, and pathways regulating the metabolic homeostasis like Type I diabetes mellitus (Table 1).

**Table 1:** Pathway analysis (DAVID) of proteins significantly elevated by pBM-MSC treatment in the kidney after extended liver resection.

Associated KEGG-Pathway	*p*-Value	Genes Involved (EntrezGeneID)
Cytokine-cytokine receptor interaction	1.8E-9	Fas ligand (356)
		RANTES (6352)
		GH (2688)
		IL-1alpha (3552)
		IL-12p70 (3593)
		IL-17A (3605)
		PDGF A/B (5154/5155)
		VEGF (7422)
MAP signaling pathway	1.2E-3	Fas ligand (356)
		FGF19 (9965)
		IL-1alpha (3552)
		PDGF A/B (5154/5155)
Pathways in cancer	2.6E-3	Fas ligand (356)
		FGF19 (9965)
		PDGF A/B (5154/5155)
		VEGF (7422)
Type I diabetes mellitus	2.9E-3	Fas ligand (356)
		IL-1alpha (3552)
		IL-12p70 (3593)
Focal adhesion	5.9E-3	PDGF A/B (5154/5155)
		Osteopontin (6696)
		VEGF (7422)
Melanoma	8.0E-3	FGF19 (9965)
		PDGF A/B (5154/5155)
Toll-like receptor signaling	1.6E-2	RANTES (6352)
		IL-12p70 (3593)
		Osteopontin (6696)

Data are taken from Fig. 7. Associated KEGG-pathways with *p*-values of significance as well as genes involved are shown.

## Discussion

The results indicate that treatment with pBM-MSCs improved liver function and signs of AKI, and thus obviously protected the kidney from damage by contributing to circulatory maintenance after extended liver resection.

Epithelial integrity is maintained by N-cadherin in proximal and by E- and N-cadherin in distal tubules. In humans, AKI induced by ischaemia or calcineurin inhibitors depleted the expression of N-cadherin while E-cadherin was unaffected^31^. Our study corroborated these findings and demonstrated that the depletion of N-cadherin was attenuated by pBM-MSCs. N-cadherin was expressed both at the cell surface and in the cytosol indicating rather the re-distribution than the transcriptional down-regulation of N-cadherin expression. Similar results were obtained for the tight junction protein ZO-1. Obviously, pBM-MSCs protected the proximal tubules from damage by the maintenance of cell-cell contacts, thus preserving epithelial integrity. This is substantiated by the alleviation of the increases in urinary NGAL and KIM-1 by pBM-MSCs. NGAL is regarded a versatile biomarker of AKI^36, 37^. It is filtered by the glomeruli and re-absorbed by the proximal tubule epithelia. Its appearance in the urine indicates the impairment of uptake consistent with the down-regulation of N-cadherin and cytoskeleton breakdown leading to the loss of polarity and barrier function in proximal tubular epithelia due to acute injury^31^. Similarly, hyponatraemia and hyperkalaemia were corrected by pBM-MSC treatment indicating the normalization of sodium re-uptake and potassium excretion. Hence, we conclude that pBM-MSCs prevented progress to AKI after major liver surgery corroborating previous results showing that MSCs avoided kidney injury induced by ischaemia-reperfusion injury in small animal models^38, 39^.

The hyperdynamic circulation is typical for patients with liver cirrhosis developing a hepatorenal syndrome (HRS)^25^. Here, we observed a similar situation after extended liver resection, which was ameliorated by pBM-MSC treatment. The fall of the peripheral blood pressure and hyponatraemia, both observed in our study, would normally activate the renin-angiotensin system to re-adjust the physiological blood pressure. Since albumin was decreased in the serum, the capacity of the liver to synthesise sufficient amounts of angiotensinogen may also be severely impaired after extended resection. Yet, the provision of angiotensinogen by the liver to maintain the renin/angiotensin axis seemed sufficient as evidenced by the rise in aldosterone; this, however, was not effective to sustain blood pressure regulation.

To elucidate the potential mechanism mediating blood pressure maintenance by pBM-MSCs, we determined cytokines in serum and kidney. There was a significant increase in PDGF in the pBM-MSC-treated animals. The Pringle’s manoeuvre during extended liver resection caused a significant drop of systemic perfusion rendering the organism with a high demand of systemic blood pressure regulation. This might be hardly achieved in control animals due to hepatic and renal impairment, but the elevation of PDGF in animals treated with pBM-MSCs may account for the relatively stable perfusion parameters indicating adequate organ perfusion. PDGF has been reported to act like angiotensin II^40^ regulating MAP maintenance by augmenting vasoconstriction and total peripheral resistance. PDGF may bypass the renin-angiotensin system and act as a potent vasoconstrictor to stabilize blood pressure and sufficient organ perfusion. A more detailed description of this hypothesis is presented in Supplementary Fig. S1. Our assumption is supported by studies showing that PDGF stabilised the MAP significantly by improving liver and kidney perfusion in a rat model of hemorrhagic shock^41^. Most renal cells express parts of the PDGF signaling chain, either the ligands or the receptors. As a response to injury, they are differentially expressed locally and regulate multiple biological processes involved in injury and wound healing like inflammation, cell proliferation and migration as well as haemodynamic homeostasis^42^. Here, we saw significant up-regulation of PDGF in the serum indicating rather a systemic than a local mode of increase. Since it has been shown that platelets supported liver regeneration after partial hepatectomy^43, 44^, we may hypothesise that the increase observed here might be platelet-derived. This, however, is speculative at the moment and needs further experimental proof.

We identified 14 proteins, which were up-regulated by pBM-MSCs in the kidney. Osteopontin supported repair of proximal tubule cells after ischaemic injury by suppressing cytotoxic macrophages^45–47^, in line with the decrease of NGAL in the urine by pBM-MSC treatment in our study. Further, VEGF was up-regulated in the kidney parenchyma. Thus, together with the increase in peripheral vasoconstriction stimulated by PDGF as discussed above, the elevation of VEGF might protect the kidney parenchyma from ischaemic insult through its vasodilatory mode of action along the VEGF-NO-axis^48, 49^. This corroborates previous results showing that MSCs elevated VEGF in rat kidney parenchyma after ischaemic injury^50^, and that they enhanced VEGF in the glomerulus after induction of nephropathy in the rat^51^. Porcine BM-MSCs also increased RANTES, MIP3alpha, IL-17A and IL-12p70 reported to be associated with kidney impairment after systemic immunological overreaction^52^. The initial pro-inflammatory response in the kidney represents a first line defense against damage thereby fostering early tissue repair^18^. Consistently, RANTES, released by the tubular epithelial cells, together with Angiotensin II receptor activation led to macrophage recruitment^53^. According to our network analysis, IL-1A might activate DPPIV to truncate RANTES into an inactive form^54^, thus substantiating the immune-modulatory properties of pBM-MSCs in the kidney. Finally, the elevation of GH and FGF19 by pBM-MSCs might support kidney repair, since these factors re-established polarity and function of tubule cells^35^. Though we need experimental proof of the mechanisms described, the pathways involved in pBM-MSC action as identified here comprised the regulation of inflammation and tissue homeostasis, aiming to rebuild kidney architecture and function impaired after extended liver resection. This corroborates previous results in the rat, where ischaemia/reperfusion in the liver caused oxidative stress, inflammation and tissue damage in the kidney. IL-18BP improved the kidney^55^, thus substantiating that MSC-mediated paracrine mechanisms might contribute to kidney repair after insult caused by hepatic ischaemia/reperfusion in the context of liver resection.

In summary we conclude that porcine BM-MSCs ameliorated signs of AKI after extended liver resection. They elevated circulatory PDGF, which might have stabilised peripheral haemodynamics, and thus adequate organ perfusion. In the kidney, pBM-MSCs might improve the intra-renal blood flow by VEGF-mediated vasodilation. Finally, pBM-MSCs promoted tissue repair by the elevation of cytokines and growth factors to modulate local immune reactions and promote tissue remodeling.

Acute kidney injury is one of the major complications associated with extended partial liver resection increasing post-surgery morbidity and mortality. Major factors contributing are ischaemic insult of the kidney due to blood loss and hypoperfusion as well as liver failure due to functional impairment. In the pig model presented here, both renal and hepatic dysfunctions were obvious after extended liver resection. Treatment with pBM-MSCs appears to be a promising novel strategy to treat AKI in the context of acute liver failure after extended resection.

## Methods

### Animals

All animal experiments were approved by the federal state authority of Saxony and were in compliance with the animal welfare act. Adult male German landrace pigs (bodyweight: 25–30 kg) were obtained from the farm product company Kitzen (Kitzen) and were housed at the medical experimental center at the University of Leipzig (MEZ Leipzig). Animals were kept under a 12 h circadian rhythm at 25 °C receiving a standard pig diet for at least 3 days. 24 h before surgery, animals were starved and 5 animals each were randomly divided into 2 groups. In addition to extended liver resection, one group received a central venous infusion of Ringer solution (control group). The second group (pBM-MSC group) 1 × 10^8^ hepatocytic differentiated pBM-MSCs. Sham animals (untreated, without liver resection) were performed for reference. The method for the isolation, hepatocytic differentiation and biochemical characterization of porcine MSC from bone marrow has been described in detail previously^56^, and summarised in the Supplementary Material. MSCs were used after hepatocytic differentiation, because we demonstrated that they secreted a significantly larger panel of hepatotropic factors as compared to undifferentiated MSCs^57^.

### Extended liver resection

**Figure 8:**
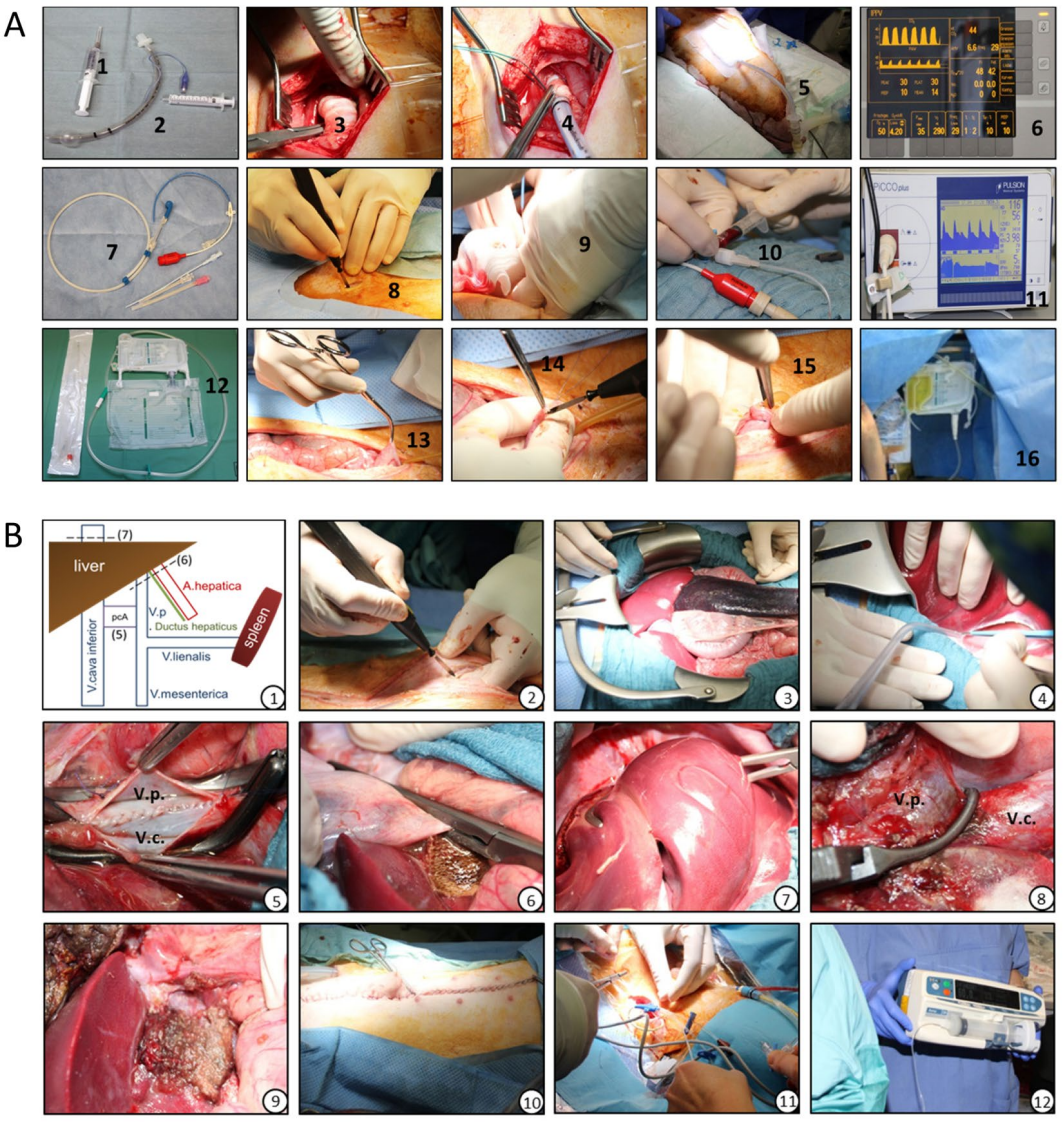
(**A**) Preparation of catheters for mandatory ventilation, blood sampling and blood pressure measurement, and urine sampling. *1*–*6 Tracheotomy:* 1 = Xylonest; 2 = oral airway; 3 = cartilage of the trachea; 4 = placing the oral airway into the trachea; 5 = connecting with mandatory ventilation; 6 = monitoring of ventilation parameters. *7*–*11 Arterial aditus:* 7 = arterial catheter; 8 = preparation of *A. femoralis*; 9 = cannulation of the *A. femoralis*; 10 = blood sampling; 11 = blood pressure measurement. *12*–*16 Suprapubic catheter:* 12 = catheter and Ureofix®; 13 = preparation of bladder; 14–15 = placing the suprapubic catheter; 16 = urine sampling. (**B**) Summary of surgical procedures for 70% liver resection. (1) Schematic overview of clamping during liver resection. After opening of the abdominal cavity (2), the retractor was placed (3) and the liver lobes were mobilized (4). After placing the portocaval anastomosis (5), the *Lig. hepatoduodenale* (6) and the *V. cava inferior* (7) were clamped. In addition to the removal of the left and medial liver lobes, the portocaval anastomosis was occluded (8). (9) shows the liver remnant representing the right lateral lobe. The abdominal cavity was closed (10) and either Ringer solution or 1 × 10^8^ pBM-MSCs (after hepatocytic differentiation) were infused through a central venous catheter (11 and 12). V.p. = *Vena portae*; V.c. = *Vena cava inferior*; pcA = portocaval anastomosis.

Surgery was performed according to Arkadopoulos^28^, with modifications. After premedication (ketamine 15 mg/kg BW, atropine 0.01 mg/kg BW, and midazolam 0.5 mg/kg BW), pigs received a continuous total venous anesthesia and analgesia (midazolam 1 mg/kg BW/h, sufentanil 0.5 μg/kg BW/h, ketamine 5–15 mg/kg BW/h, pancuronium bromide 80 μg/kg BW/h). In addition, a tracheotomy was performed for mandatory ventilation (Fig. 8A, 1–6). The A. femoralis was cannulated for blood sampling and for invasive blood pressure measurements using the PiCCO-system (Pulsion Medical Systems, Germany) (Fig. 8A, 7–11). Control solution (Ringers acetate, Gelafusal® (Serumwerk Bernburg AG, Germany) and dextrose 5%; 6:3:1) or pBM-MSCs were infused through a central venous catheter placed in the V. jugularis interna. Urine was collected from a suprapubic catheter placed and connected to the Ureofix®-System (B. Braun Melsungen AG, Germany) (Fig. 8A, 12–16). For liver resection, the abdominal cavity was opened (60 cm incision along the Linea alba), the retractor placed and liver lobes were mobilized. In addition to a portocaval anastomosis, the Lig. hepatoduodenale was occluded proximally to the liver hilus (Pringle’s manoeuvre). During 150 min of warm ischemia, the left and medial liver lobes were resected (70% of total liver mass) (Fig. 8B). Finally, the portocaval anastomosis was occluded and the closure of the Lig. hepatoduodenale released. To maintain cardiovascular performance, animals were supplemented with Norepinephrine Hydrochloride as appropriate.

### Blood and urine sample analysis

Before, at zero-time and at 3, 6, 9, 12, 18, and 24 hours after liver resection, blood and urine samples were collected from the arterial and the suprapubic catheters. Aspartate and alanine aminotransferase (AST, ALT), ammonia, total bilirubin, albumin, potassium, sodium and creatinine as well as aldosterone were measured in the blood serum at the Department of Diagnostics (University Hospital Leipzig). NGAL and KIM-1 were measured in urine or blood serum using the porcine-specific NGAL- (Dianova GmbH) or the KIM-1 ELISA (Elabscience) according to the manufacturers´ instructions. The clearance of indocyanine green was measured using the commercial LIMON system (Pulsion Medical Systems).

### Proteome and transcriptome array analysis

Cytokines were detected in kidney tissue and serum samples using the Proteome Profiler^TM^ Human Cytokine Array Kit (R&D systems). The antibodies on the array were anti-human specific, hence probably not detecting changes in the pig unequivocally. Relative signal intensity was quantified with ImageJ and the additional plug-in “array analyzer” (National Institutes of Health, NIH). Proteins significantly different between control and pBM-MSC groups were entered into the online STRING-platform (Known and Predicted Protein-Protein Interactions; http://string-db.org/) to delineate potential interactions. Predicted pathway analysis was performed using the DAVID Bioinformatics Resources 6.7 database (https://david.ncifcrf.gov/)^58, 59^, and associated with respective KEGG-pathways (http://www.genome.jp/kegg/pathway.html).

Sample processing for transcriptomic analyses was performed by the Affymetrix Service Provider and Core Facility, “KFB - Center of Excellence for Fluorescent Bioanalytics” (Regensburg, Germany; www.kfb-regensburg.de).

### Histological procedures and RT-PCR

HE and ZO-1 staining, and co-stainings of cadherins were performed according to standard protocols as outlined in the Supplementary Material.

### Statistics

Data were analyzed assuming normal distribution and homoscedasticity by the Shapiro–Wilk test. As most of the data did not match these criteria, general linear models (GLM) were used to test for significant differences between treatment groups (control and pBM-MSCs), effect of time after liver resections and the combined effects of both. Bonferroni post-hoc tests were used for pair-wise comparisons of individual treatment groups and time points after liver resection. All statistical analyses were done using STATISTICA 8.0 (StatSoft, Tulsa, OK).

## Electronic supplementary material

Supplementary Information
