# Supplementary material for: Mesenchymal stem cells correct haemodynamic dysfunction associated with liver injury after extended resection in a pig model
Source: Sci Rep. 2017 Jun 1;7:2617. doi: 10.1038/s41598-017-02670-8 (PMC5454025; doi:10.1038/s41598-017-02670-8)
Supplement: Supplementary file 1 — Supplementary Information [file 41598_2017_2670_MOESM1_ESM.pdf]

Journal: Scientific Reports

Title: Mesenchymal stem cells correct haemodynamic dysfuntion associated with liver injury after extended resection in a pig model

Authors:

Hans-Michael Tautenhahn, Sandra Brückner, Christiane Uder, Silvio Erler, Madlen Hempel, Martin von Bergen, Janine Brach, Sandra Winkler, Franziska Pankow, Claudia Gittel, Manja Baunack, Undine Lange, Johannes Broschewitz, Matthias Dollinger, Michael Bartels, Uta Pietsch, Kerstin Amann, Bruno Christ

Correspondence and requests:

E-mail: bruno.christ@medizin.uni-leipzig.de

Department of Visceral, Transplantation, Thoracic and Vascular Surgery

University Hospital Leipzig AöR

Liebigstraße 21

D-04103 Leipzig

Germany

Phone: +49(0)341 9713555; Fax: +49(0)341 9713559

Supplementary Material

Isolation and hepatocytic differentiation of mesenchymal stem cells from porcine bone marrow (pBM-MSC)

Mesenchymal stem cells were isolated from bone marrow of adult German landrace pigs using standard collagenase (NB4G, Serva GmbH, Germany) and the digestion protocol essentially as described previously [1]. The stem cell isolates were routinely analyzed by flow cytometry detecting surface markers CD14, CD29, CD44, CD45, CD90 and CD105 at the core unit “Fluorescence Technologies” of the Interdisciplinary Centre for Clinical Research (IZKF), Leipzig, using the BD LSRII and BD FACS Diva v.6.1.3 software. Thereafter, MSC were differentiated into hepatocyte-like cells using hepatocyte growth medium (HGM supplemented with HGF and EGF) essentially as described previously.[1] Since we anticipated that paracrine mechanisms might be involved in MSC action, we used hepatocytic differentiated MSC, which secreted to higher amounts and much more proteins than undifferentiated cells.[2]

Histological procedures

HE staining

2h h after liver resection, kidney tissue was embedded with paraffin and 1-3 µm sections stained according to standard protocols with haematoxylin for 2 min and with eosin for 5 min.

E-cadherin and N-cadherin co-staining, ZO-1 staining

After dewaxing, the paraffin slices were incubated with TRIS-buffer (10 mM, pH 9.0) followed by incubation in blocking solution (5% goat serum) for 20 min, and in BSA-blocking solution (5% BSA and 0.5% Tween 20 in phosphate-buffered saline (PBS)) for 60 min. Thereafter, slices were incubated with the primary anti-E-cadherin and anti-N-cadherin antibodies or the ZO-1 antibody overnight at 4°C. After three washings with PBS, the first secondary antibody Cy3 was applied for 70 min at room temperature. After three additional PBS washings, the second secondary antibody AlexaFluor488 was applied for 70 min at room temperature. After another washing with PBS for three times, slices were counterstained with DAPI solution (Roth GmbH, Germany) and embedded in glycerin solution (50%; Roth GmbH, Germany) for microscopic analysis.

Antibody	Species	Reactivity	Dilution	Company	Catalog number
E-cadherin	mouse	human, mice, rat, pig	1:200	BD	610182
N-cadherin	rabbit	human, mice, rat, pig	1:200	Millipore	04-1126
ZO-1	Rabbit	Human, dog, rabbit, mouse	1:150	InVitrogen	40-2200
Cy3	goat	mouse	1:200	Dianova	115-165-003
AlexaFluor 488	goat	rabbit	1:200	Life Technologies	A11008

Primary and secondary antibodies used for immunohistochemistry of cadherins.

RT-PCR

Total RNA was extracted using a standard Trizol protocol supplied by the provider. The reverse transcriptase (RT) reaction was performed with the Maxima-H-Minus-First-Strand cDNA synthesis kit (Thermo-Fisher) as specified by the manufacturer. The resulting cDNA was used as the template for PCR reactions (pre-degeneration: 95°C for 5 min; degeneration: 95°C for 30 s; annealing: 57.5°C for 30 s; extension: 72°C for 45 s; 35 cycles; terminal extension: 72°C for 4 min). PCR products were analyzed by agarose gel electrophoresis and relative intensity of specific bands was quantified using ImageJ (National Institutes of Health, NIH).

**gene** *(NCBI reference sequence)*	**fwd 5´- 3´**	**rev 5´- 3´**
**PPIA** *(NM_008907.1)*	CCCACCGTGTTCTTCGACA	GTAAAGTCACCACCCTGGCA
**caspase 3** *(NM_214131.1)*	GGATTGAGACGGACAGTGGG	CCGTCCTTTGAATTTCGCCA

**caspase 8** *(NM_001031779.2)*	GGGATGGCCACTGTGAACAA	CTTCACCCCTGGGACATCTT
**N-cadherin** *(XM_013996117.1)*	ACAATGATCCCACAGCTCCG	GCCCCCAATCGTTCAGGTAA
**ZO-1** *(XM_013993251.1)*	CAACAGCATCCTCCCACCTT	TTAGGATCACCCGACGAGGA
**occuldin** *(NM_001163647.2)*	TGCACCCTCCAGATTGGC	AAAGCATTGGTCGAATGGGC
**CK18** *(XM_005652579.2)*	CACCTCAGTGGTCACCTCAC	CTCCAAGGTGGCCTTCAGAT
**IL-18** *(XM_005667326.2)*	GCTGCTGAACCGGAAGACAA	TCCGATTCCAGGTCTTCATCG

Primer pairs used for RT-PCR analyses.

Hypothesis - Support of blood pressure homeostasis by PDGF elevated by pBM-MSC treatment

There was a very obvious increase in PDGF in the serum of animals treated with pBM-MSCs as compared with controls. Together with the observation that the MAP as well as the cardiac output was more stable in animals receiving pBM-MSCs as compared to the controls, and the fact that the kidneys are major players in blood pressure regulation, we hypothesize the following. The Pringle's manoeuvre during extended liver resection caused a significant drop of systemic perfusion rendering the organism with a high demand of systemic blood pressure regulation. This might be hardly achieved in control animals, but the elevated amount of PDGF in blood serum of animals treated with pBM-MSCs may account for the relatively stable perfusion parameters indicating adequate whole body perfusion including the kidneys. PDGF has been reported to act like Angiotensin II, which is the major regulator of the Renin-Angiotensin system mediating vasoconstriction and elevation of TPR (total peripheral resistance) to maintain a constant MAP. Angiotensinogen, secreted by the liver, is converted

to Angiotensin I by Renin, which is released by cells of the juxtaglomerular apparatus after sensing of systemic blood pressure fall caused by the surgical intervention. Angiotensin I is converted to angiotensin II by ACE (Angiotensin converting enzyme). Thus, the functional impairment of the liver after extended resection is likely to impair also the Renin-Angiotensin system due to the decreased production of Angiotensinogen. PDGF, elevated by the treatment with pBM-MSC, may bypass the Renin-Angiotensin axis and act like Angiotensin II as a potent vasoconstrictor to stabilize blood pressure and sufficient organ perfusion (Supplementary Fig. S1)

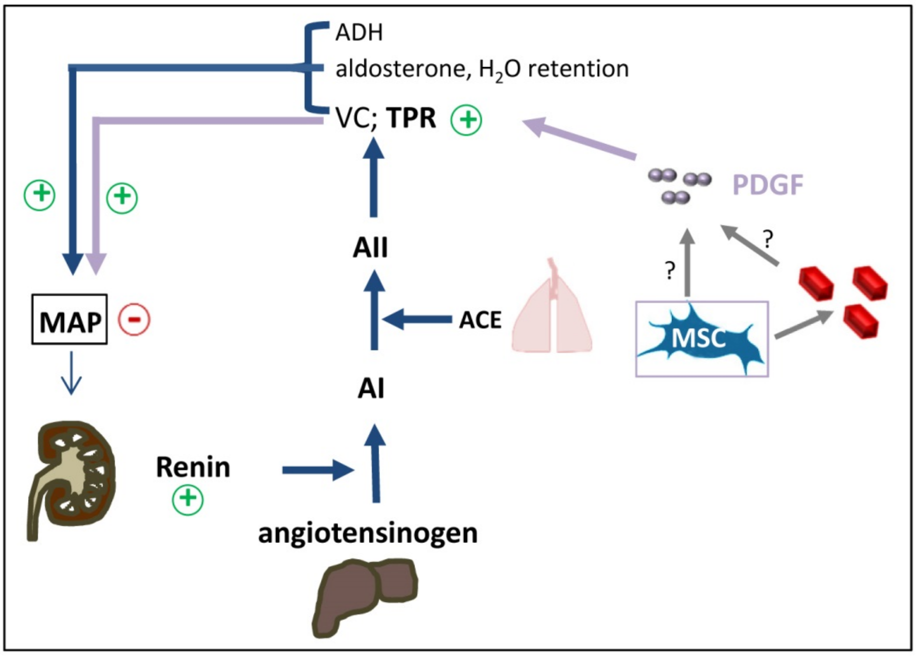

Supplementary Figure S1. If the systemic pressure (MAP = mean arterial pressure) is decreased, renin is released by the kidney parenchyma and converts angiotensinogen, provided by the liver, into angiotensin I (AI). In addition, the angiotensin converting enzyme (ACE), provided by the lungs, converts angiotensin I into angiotensin II (AII). Angiotensin II elevates ADH (antidiuretic hormone), aldosterone production and the TPR (total peripheral resistance) by vasoconstriction (VC). Thus, the MAP is maintained physiologically by the renin-angiotensin system. In case of liver damage, the provision of angiotensinogen is impaired. PDGF (platelet derived growth factor), secreted by pBM-MSCs, or after stimulation of other cells, leads to vasoconstriction in a similar manner like angiotensin II, resulting in elevation of TPR and MAP bypassing the renin-angiotensin system.

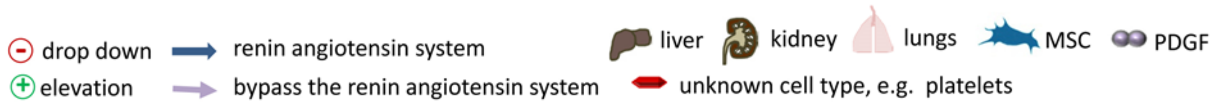

References

1 Bruckner, S. *et al.* A fat option for the pig: hepatocytic differentiated mesenchymal stem cells for translational research. *Exp Cell Res* **321**, 267-275, doi:10.1016/j.yexcr.2013.10.018 (2014).

2 Winkler, S. *et al.* Identification of Pathways in Liver Repair Potentially Targeted by Secretory Proteins from Human Mesenchymal Stem Cells. *Int. J. Mol. Sci.* **17**, 1099, doi:10.3390/ijms17071099 (2016).
